# Age-dependent prognostic significance of neutrophil-to-HDL ratio in hepatocellular carcinoma patients

**DOI:** 10.3389/fonc.2025.1620017

**Published:** 2026-01-07

**Authors:** Qingjun Li, Ting Wang, Xiangjun Qian, Hang Xing, Zhongyi Li, Zhengzheng Wang, Shuaiya Ma, Bo Meng, Xianzhou Zhang, Feng Han, Hao Wang, Jinxue Zhou, Yongmei Li, Hao Zhuang

**Affiliations:** 1 Department of Hepatobiliopancreatic Surgery, The Affiliated Cancer Hospital of Zhengzhou University & Henan Cancer Hospital, Zhengzhou, China; 2 Department of Radiation Oncology, The Affiliated Cancer Hospital of Zhengzhou University & Henan Cancer Hospital, Zhengzhou, China; 3 Division of Cardiothoracic Surgery, Rhode Island Hospital, Alpert Medical School of Brown University, Providence, RI, United States; 4 The Affiliated Cancer Hospital of Zhengzhou University & Henan Cancer Hospital, Zhengzhou, China; 5 Department of Pathogen Biology and Department of Genetics, School of Basic Medical Sciences, Tianjin Medical University, Tianjin, China

**Keywords:** hepatocellular carcinoma, biomarker, neutrophil-to-HDL ratio (NHR), inflammatory-lipid markers, aging

## Abstract

**Background:**

Hepatocellular carcinoma (HCC) is a highly heterogeneous malignancy, with variability in molecular features, clinical presentations, and treatment responses. Postoperative recurrence and disease-free survival (DFS) are important prognostic indicators for patient outcomes. The neutrophil-to-HDL ratio (NHR) is recognized as an inflammatory-lipid marker, however its age-dependent predictive value in HCC remains unclear and is not established in clinical practice. Therefore, we aimed to evaluate the prognostic significance of NHR in HCC patients undergoing surgical resection, with a focus on its age-dependent effects.

**Methods:**

We retrospectively analyzed 121 HCC patients undergoing surgical resection and randomly divided them into training (n = 95) and validation (n = 26) cohorts. Multivariate logistic regression, Receiver operating characteristic (ROC) analysis, and nomogram construction were used to evaluate the prognostic significance of NHR, with age-stratified analyses conducted to explore its differential effects.

**Results:**

In the training cohort, both univariate and multivariate analysis identified NHR and age as statistically significant prognostic factors for HCC recurrence (*P* < 0.05). Age-stratified analysis further demonstrated that the prognostic value of NHR was significant in older patients (OR = 0.087, 95% CI: 0.009 - 0.835, *P* = 0.034), but not in younger patients. ROC analysis indicated good predictive performance for both NHR (AUC = 0.609) and age (AUC = 0.655). Similar trends were observed using the validation dataset.

**Conclusions:**

In this cohort of 121 HCC patients, NHR showed a potential association with prognosis in older patients; However, these findings are preliminary due to limited sample size and lack of stratified analyses by disease stage and prior treatment. Future studies should validate these findings in larger, well-characterized cohorts and investigate underlying mechanisms.

## Introduction

Hepatocellular carcinoma (HCC) ranks as the sixth most prevalent cancer globally and is the third leading cause of cancer-related deaths (1–3). Despite advances in both surgical and systemic treatments, the prognosis of HCC remains poor due to its biological heterogeneity and high recurrence rates. Five-year survival rates vary widely, from approximately 70% for early-stage disease to less than 16% in advanced stages (4, 5). Accurate prognostic markers are crucial for guiding individualized treatment strategies and improving patient outcomes. Traditional prognostic factors, such as tumor size, stage, and differentiation, often fail to fully capture the complexity of HCC, highlighting the need for more comprehensive and accessible biomarkers to guide clinical decision-making and facilitate the development of individualized treatment strategies.

Systemic inflammation and lipid metabolism dysregulation in cancer progression. Individual markers of pro-inflammatory activity, such as neutrophil count (6, 7), and lipid parameters with anti-inflammatory properties, like high-density lipoprotein cholesterol (HDL-C) (8, 9), have been independently associated with tumor progression and survival. However, single markers may not fully capture the complex interplay between inflammation and lipid metabolism in cancer. The neutrophil-to-HDL ratio (NHR) integrates these two components, reflecting systemic inflammation and metabolic dysregulation more comprehensively than traditional single markers. NHR has demonstrated significant prognostic value across various fields as a biomarker of systemic inflammation and oxidative stress (10–12). NHR effectively predicts all-cause and cardiovascular mortality in the general population (10) and individuals with prediabetes (11). NHR is also reported as a prognostic cancer marker for HCC. Combining NHR with end-stage liver disease scores helps clinicians identify high-risk patients early, facilitating timely and optimized management strategies (12).

Age is a critical factor influencing cancer progression and prognosis through complex biological mechanisms (13–15). Older HCC patients experience immune senescence and lipid metabolism changes, such as impaired neutrophil function and decreased HDL-C levels (16, 17), which may amplify the prognostic relevance of inflammatory-lipid markers like NHR. These age-related changes also contribute to treatment effectiveness and inflammatory responses (13, 18). Consequently, understanding how age modifies the predictive value of NHR is essential for improving risk stratification in elderly HCC patients – a relationship that, to date, remains largely unexplored.

This study focuses on the novel role of the NHR as an age-stratified prognostic marker, aiming to address a critical gap in current models. By evaluating the prognostic value of NHR in HCC patients and exploring its potential age-specific effects, we aim to provide a foundation for more personalized and biologically informed therapeutic strategies.

## Methods

A total of 121 HCC patients who underwent surgical resection at Henan Cancer Hospital, China between 2011 and 2012 were included. During this period, neoadjuvant and adjuvant therapies were not recommended by the Chinese liver cancer guidelines, and none of the patients received such treatment, ensuring therapeutic homogeneity within the cohort. Patients were classified as having a “better prognosis” if they remained disease-free for longer than the mean DFS of the cohort and/or had no recurrence at last follow-up. Those with recurrence and/or DFS less than or equal to the mean were classified as having a “worse prognosis”. Among the 121 patients, 95 were randomly assigned to the training dataset for the development the prognostic model, with the remaining 26 formed the validation dataset. Standardized postoperative follow-up (every 3 to 6 months) and consistent recurrence management protocols minimize treatment-related confounders, ensuring the reliability of the NHR analysis.

In the current analysis, tumor differentiation states were categorized according to the Barcelona Clinic Liver Cancer (BCLC) staging system, where lower tumor differentiation was defined as 0 to B stage cases, while higher stages represented others. Patients were stratified into younger and older groups based on the mean age (55.6 ± 8.7 years) of the whole cohort. The lipid-inflammatory profiles in this analysis included both original and derivative indicators. The original indicators comprised low-density lipoprotein cholesterol (LDL-C), high-density lipoprotein cholesterol (HDL-C), total cholesterol (TC), very-low-density lipoprotein cholesterol (VLDL-C), neutrophils, lymphocytes, white blood cells (WBC), and platelets. The derivative indicators consisted of non-HDL, the LDL-to-HDL ratio, the TC-to-HDL ratio, RC (TC-HDL-LDL), and PWR (Platelet-to-WBC ratio). Additionally, the analysis included PLR (platelet-to-lymphocyte ratio), NLR (neutrophil-to-lymphocyte ratio), NHR (neutrophil-to-HDL ratio), and the lymphocyte-to-HDL ratio (LHR). Also, a cutoff of 55 years was used for age stratification in this manuscript, based on the mean age of our cohort (55.6 ± 8.7 years). This approach aligns with similar stratification methods in comparable HCC studies and offers balanced subgroups for our analysis.

### Statistical analysis

Double entry was utilized to ensure the accuracy of the data. Normality was evaluated using the univariate and multivariate coefficients of skewness and kurtosis. The comparison of quantitative and categorical data was carried out using ANOVA and the Chi-square/Fisher’s exact test. Non-parametric analysis was applied to the data with a non-normal distribution. Logistic regression models were employed to establish the predictive model for HCC recurrence; subsequently, the nomogram was presented to detail the multivariate regression results and the interaction among predictive factors. To further evaluate the predictive performance of NHR and age as prognostic markers, receiver operating characteristic (ROC) analysis was performed. Considering the potential confounding effect of age, additional stratified analyses by age groups were conduct to assess the consistency of the association between NHR and postoperative recurrence across different age strata. A *P*-value of less than 0.05 was considered statistically significant. SPSS 16.0 statistical software and R 4.4.2 were utilized for data processing.

## Results

### General characteristics of the study population

The baseline characteristics of the 121 HCC patients are summarized in **Table 1**. The demographic and clinical variables distribution, including age, gender, tumor differentiation, tumor number, and operation duration, showed no significant differences between patients with better and worse prognoses (*P* > 0.05). Tumor differentiation and number were evenly distributed across prognosis groups, indicating a comparable baseline for further analysis.

**Table 1:** General characteristics between different prognosis groups.

Variable		*P* for tests of normality (Kolmogorov-Smirnova)	Better prognosis	Worse prognosis	*χ²/F/Z*	*P*
Age		0.200	57.50 ± 6.00	53.75 ± 11.54	1.391	0.242
Tumor differentiation	Lower		11	27	0.142	0.110
	higher		2	32		
Tumor number		<0.001	1.00 (1.00, 5.00)	1.00 (1.00, 1.00)	-0.585	0.558
Operation duration (min)		0.200	205.01 ± 101.22	184.62 ± 79.14	0.690	0.409

Mean (SD); n (%).

### Inflammatory-lipid profiles and prognosis

The inflammatory-lipid profiles of patients demonstrated significant differences between better and worse prognosis groups (**Table 2**). HDL-C levels were significantly lower in patients with worse prognoses (0.79 ± 0.08 mmol/L *vs.* 1.09 ± 0.23 mmol/L, *P* = 0.001), while VLDL-C levels were significantly higher in this group (0.22 [0.17–0.25] mmol/L *vs.* 0.11 [0.02–0.17] mmol/L, *P* = 0.005). Other markers, including NLR and PLR, showed no statistical significance (*P* > 0.05). Although NHR levels were higher in the worse prognosis group, they did not reach statistical significance in the overall cohort.

**Table 2:** Characteristics of inflammatory-lipid profiles between different prognosis groups in the training dataset.

Variable	*P* for tests of normality (Kolmogorov-Smirnova)	Better prognosis	Worse prognosis	*χ²/F/Z*	*P*
WBC	0.200	6.19 ± 1.76	6.45 ± 2.65	0.114	0.736
Neutrophils	0.200	3.88 ± 1.27	4.21 ± 2.17	0.297	0.587
Lymphocyte	<0.001	1.96 (1.13, 2.61)	1.65 (1.07, 2.18)	-1.222	0.222
Platelet	0.200	91.40 ± 24.43	88.72 ± 10.92	0.035	0.851
PLR	0.200	99.82 ± 16.35	117.89 ± 8.60	0.805	0.372
NLR	0.200	2.29 ± 0.37	3.07 ± 0.35	0.997	0.321
PWR	0.200	29.62 ± 3.60	29.59 ± 1.70	0.001	0.996
TC	0.200	3.05 ± 0.83	3.57 ± 0.92	2.086	0.153
HDL-C	0.200	0.79 ± 0.08	1.09 ± 0.23	11.402	**0.001**
LDL-C	0.200	1.97 ± 0.73	2.46 ± 0.76	2.563	0.114
VLDL-C	<0.001	0.11 (0.02, 0.17)	0.22 (0.17, 0.25)	-2.778	**0.005**
LDL-HDL-Ratio	0.200	2.51 ± 0.40	2.37 ± 0.13	0.118	0.732
TC-HDL-Ratio	0.200	3.88 ± 0.47	3.44 ± 0.16	0.744	0.391
Non-HDL	0.200	2.25 ± 0.32	2.51 ± 0.11	0.556	0.458
RC	0.200	0.28 ± 0.08	0.22 ± 0.02	0.995	0.323
LHR	<0.001	2.45 (1.49, 3.06)	1.41 (1.11, 2.05)	-1.825	0.068
NHR	0.200	5.26 ± 1.34	4.04 ± 2.16	2.115	0.150

Mean (SD); n (%). Bold values = p<0.05.

### Establishment and validation of the prognosis predictive model

Multivariate logistic regression identified NHR and age as statistically significant prognostic variables in the training cohort (*P* < 0.05, **Table 3**). A decreasing NHR was significantly associated with a better prognosis (OR = 0.601, 95% CI: 0.376 - 0.961, *P* = 0.034), while increasing age was predictive of worse outcomes (OR = 0.861, 95% CI: 0.761 - 0.973, *P* = 0.017). Other variables retained statistical significance in univariate analyses but presented insignificant in the multivariate model.

**Table 3:** Establishment of the prognosis predictive model by regression analysis based on all patients.

Variable	*ß*	*S.E*	*Wald*	*OR*	95% *CI for OR*	*P*
Age	0.872	0.181	23.263	2.392	1.678- 3.410	< 0.001
Tumor differentiation	1.875	0.812	5.331	6.519	1.328 - 32.007	0.021
Tumor nnumber	-0.481	0.236	4.141	0.618	0.389 - 0.982	0.042
HDL-C	2.430	0.443	30.041	11.356	4.763 - 27.076	< 0.001
VLDL-C	11.229	4.691	5.730	7.530	7.649 - 74.128	0.017
NHR	-0.508	0.239	4.516	0.601	0.376 - 0.961	0.034
Age	-0.150	0.063	5.730	0.861	0.761 - 0.973	0.017

**Figure 1:**
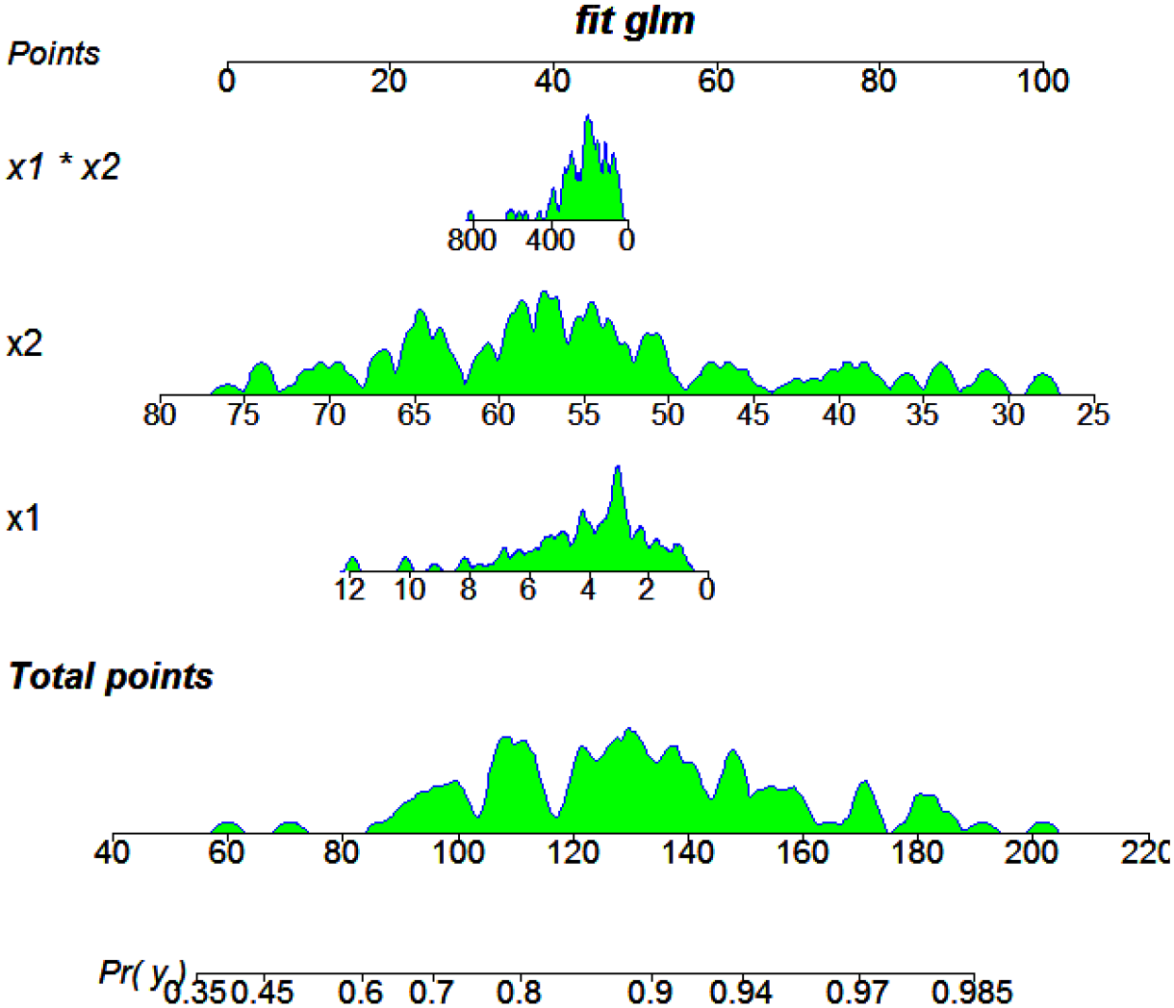
Results of multivariate regression for prognosis prediction among all patients. X1: NHR; X2: age.

Based on the regression results, we constructed a prognostic nomogram to visualize these variables’ predictive power, as shown in **Figure 1**. This model highlights the clinical applicability of integrating NHR and age into individualized prognostic assessments.

### Age-stratified analysis

To explore the age-dependent effects of NHR, the cohort was divided into two groups: younger patients (< 55 years) and older patients (≧ 55 years). The comparison of inflammatory-lipid profiles and other prognostic factors across these age groups is presented in **Table 4**. Older patients exhibited significantly higher tumor numbers (*P* = 0.010) and lower lymphocyte levels (*P* = 0.034). Platelet counts were also significantly reduced in older patients (*P* = 0.007), suggesting potential age-related differences in immune and inflammatory responses.

**Table 4:** Distribution of inflammatory-lipid profiles and other potential prognostic factors between different age groups.

Variable		Younger patients	Older patients	*χ²/F/Z*	*P*
Male:female		34:5	21:4	0.768	0.456
Tumor differentiation	Lower	16	27	0.498	0.286
	higher	17	20		
Tumor number		1.00 (1.00, 1.00)	1.00 (1.00, 1.50)	-2.587	**0.010**
Operation duration (min)		197.43 ± 97.66	186.18 ± 73.48	0.381	0.539
WBC		6.66 ± 2.44	6.01 ± 2.51	1.498	0.224
Neturophils		4.46 ± 2.28	3.96 ± 1.86	1.307	0.256
Lymphocyte		2.05 (1.22, 2.28)	1.58 (1.06, 1.94)	-2.125	**0.034**
Platelet		204.33 ± 88.42	154.03 ± 82.62	7.565	**0.007**
PLR		95.52 (53.13, 158.22)	89.94 (70.90, 130.68)	-0.785	0.432
NLR		1.89 (0.98, 4.53)	2.56 (1.90, 3.07)	-0.651	0.515
PWR		31.67 (20.45, 36.95)	26.18 (19.85, 33.35)	-1.758	0.079
TC		3.49 ± 0.64	3.58 ± 1.10	0.180	0.672
HDL-C		1.09 ± 0.21	1.02 ± 0.24	1.593	0.211
LDL-C		2.35 ± 0.57	2.49 ± 0.94	0.621	0.433
VLDL-C		0.22 (0.08, 0.22)	0.22 (0.18, 0.27)	-1.353	0.176
LDL-HDL-Ratio		2.18 ± 0.40	2.60 ± 1.37	2.880	0.094
TC-HDL-Ratio		3.26 ± 0.50	3.73 ± 1.63	2.536	0.115
Non-HDL		2.41 ± 0.55	2.60 ± 1.07	0.923	0.340
RC		0.12(0.12, 0.24)	0.19 (0.12, 0.35)	-1.578	0.115
LHR		2.05 (1.15, 2.56)	1.36 (1.10, 1.97)	-0.892	0.373
NHR		4.29 ± 2.33	4.16 ± 2.07	0.062	0.804

Mean (SD); n (%). Bold values = p<0.05.

**Figure 2:**
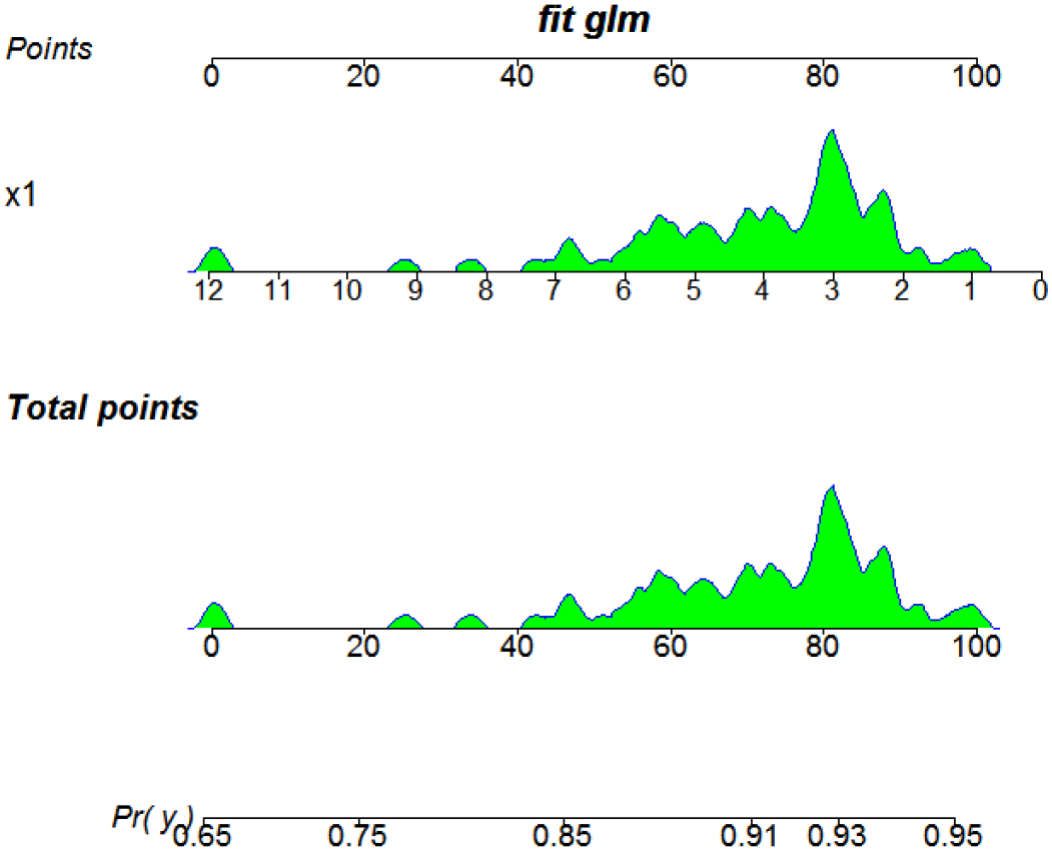
Results of the multivariate regression for predicting prognosis in older patients. X1: NHR.

As shown in **Figure 2**; **Table 5**, in older patients, NHR demonstrated a strong association with prognosis (OR = 0.087, 95% CI: 0.009 - 0.835, *P* = 0.034), visualizing this subgroup’s multivariate regression results. In contrast, no significant relationship was observed between NHR and prognosis in younger patients (*P* > 0.05). Additionally, HDL-C and tumor number retained prognostic significance in older patients but not in the younger subgroup (**Table 5**). These findings suggest that NHR has better predictive value in older HCC patients, likely due to age-related changes in immune and metabolic responses.

**Table 5:** Establishment of the prognosis predictive model through regression analysis based on different age groups.

Variable	*ß*	*S.E*	*Wald*	*OR*	*95% CI for OR*	*P*
Univariate regression analysis among younger patients						
Tumor number	1.922	0.532	13.060	6.833	2.410 - 19.375	< 0.001
HDL-C	3.417	1.070	10.203	30.471	3.744 - 247.970	0.001
LDL-C	1.689	0.553	9.318	5.412	1.830 - 16.002	0.002
VLDL-C	30.369	14.401	4.447	15.451	8.524 - 28.012	0.035
LDL-HDL ratio	1.707	0.538	10.069	5.511	1.920 - 15.814	0.002
Univariate regression analysis among older patients						
Tumor number	-0.587	0.283	4.299	0.556	0.319 - 0.968	0.038
HDL-C	2.009	0.497	16.346	7.454	2.815 - 19.737	< 0.001
NHR	0.294	0.096	9.275	1.341	1.110 - 1.621	0.002
Multivariate regression analysis among older patients						
NHR	-2.441	1.153	4.478	0.087	0.009 - 0.835	0.034

### Model validation

**Figure 3:**
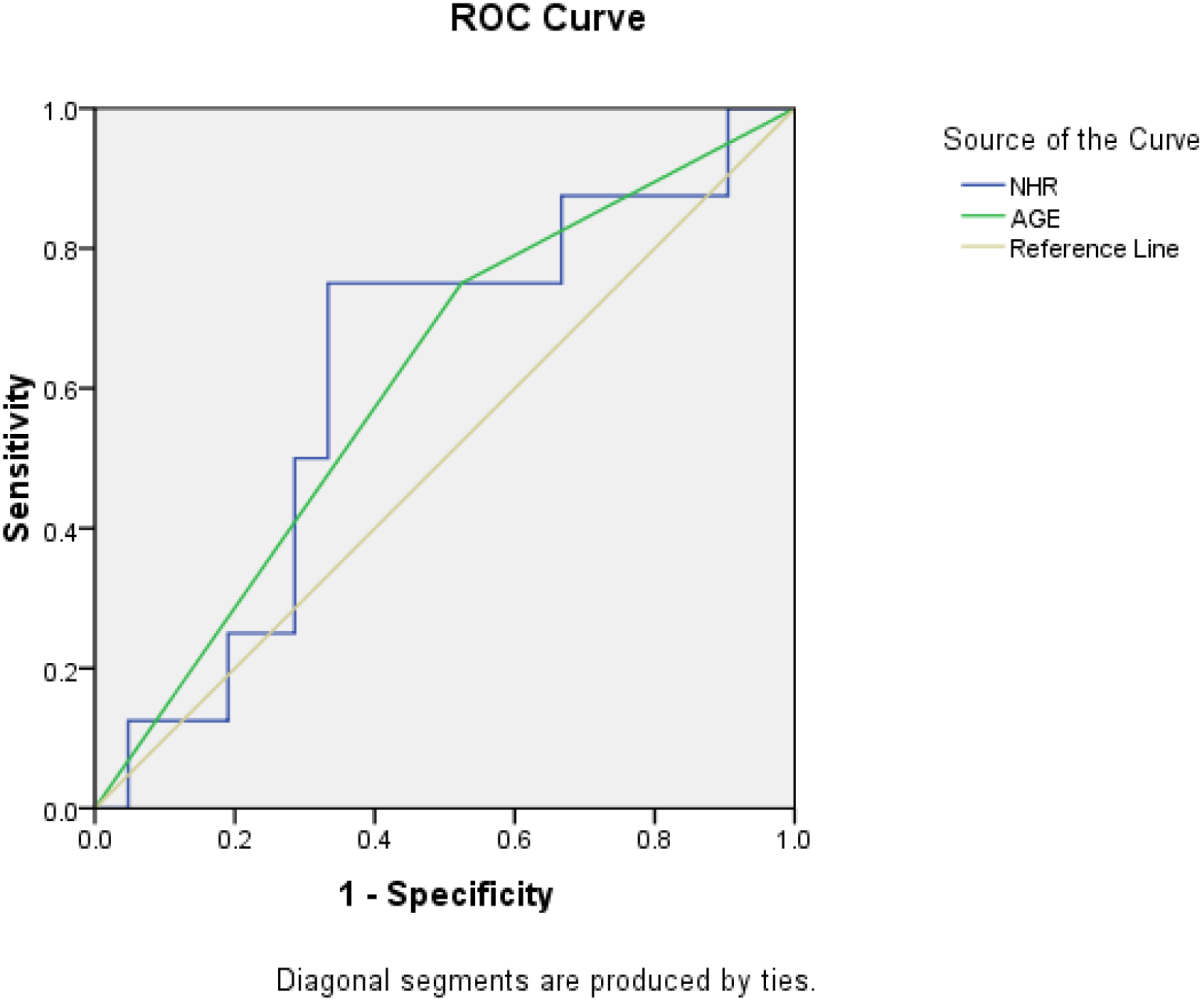
ROC for NHR and Age in predicting prognosis among HCC patients.

The independent cohort (n = 26) validated the prognostic model. ROC analysis demonstrated that NHR and age were both effective predictors of prognosis, with area under the curve (AUC) values of 0.609 and 0.655, respectively (**Figure 3**).

Collectively, this study highlights NHR as an prognostic marker in HCC, with its predictive value being particularly pronounced in older patients. The nomogram integrating NHR and age provides a practical tool for individualized risk assessment. Additionally, the age-stratified analysis underscores the importance of considering demographic factors, such as age, when evaluating prognosis in HCC patients.

## Discussion

This study demonstrated that the NHR is a significant prognostic factor in HCC patients, with its prognostic impact showing age-dependent effect. Specifically, patients with a DFS longer than the mean value of the current and/or no recurrence at the last follow-up were classified as having a better prognosis. Our findings suggest that integrating inflammatory-lipid markers into personalized prognostic models could improve HCC management. Specifically, NHR demonstrated age-dependent prognostic value, with stronger predictive power in older patients, likely due to age-related immune and metabolic changes. In older patients immune senescence, characterized by reduced adaptive immunity and heightened systemic inflammation (13, 14), may amplify the influence of inflammatory markers. Furthermore, age-related changes in lipid metabolism, such as decreased HDL-C levels, could enhance the prognostic relevance of NHR in this population. Notably, the nomogram incorporating NHR, age, and other clinical factors showed strong predictive accuracy and calibration, indicating its potential clinical utility. Compared to traditional inflammatory markers like NLR, NHR uniquely combines inflammatory and lipid components, providing a more comprehensive representation of systemic metabolic states relevant to HCC progression.

The NHR has emerged as a new biomarker reflecting the relationship between inflammation and lipid metabolism in cancer progression, with different age-dependent effects mediated through various molecular mechanisms. Literature suggests the inflammation-lipid crosstalk in aging, such as NHR, might integrate neutrophil-driven inflammation, including IL-6 and oxidative bursts, with HDL’s anti-inflammatory properties (e.g., cholesterol efflux, paraoxonase-1 activity), while aging disrupts this balance. HDL functionality declines while neutrophil activation increases, thus worsening inflammation (19–21). Additionally, metabolic dysregulation varies among age groups. HDL was found to suppress neutrophil extracellular traps (NETs) via apoA-I, maintaining vascular homeostasis in young populations, but HDL dysfunction (e.g., glycation in diabetes) fails to inhibit NETosis, increasing endothelial damage and thrombosis risk during aging (19–23).

Building on these findings, our results highlight the potential of NHR to address key gaps in current models by exploring its distinctive age-dependent effects. Most existing prognostic frameworks tend to generalize across populations, often neglecting the influence of age-specific factors (12, 15, 23–25). By highlighting the age-dependent prognostic relevance of NHR, our study underscores the necessity of incorporating demographic and biological factors, including age, into personalized prognostic assessments. This age-dependent insight not only refines risk stratification but also suggests that incorporating markers like NHR into prognostic models could enable more personalized and biologically informed treatment strategies, addressing the unmet clinical needs of diverse patient populations.

The clinical implications of this study are twofold. First, for older HCC patients, NHR might offer a readily available biomarker and cost-effective tool that can be easily integrated into routine clinical practice to enhance risk stratification, informing individualized follow-up and treatment strategies. Second, the age-dependent findings underscore the need for personalized approaches that account for demographic and biological heterogeneity in HCC patients. For example, interventions targeting neutrophil activation or promoting HDL-mediated lipid transport could improve outcomes, particularly in older patients with elevated NHR.

The NHR is an easily accessible clinical biomarker obtained from routine blood tests during standard patient care. Our current study focuses on validating NHR’s prognostic value due to its practical advantages in clinical workflows, which require no additional invasive procedures beyond standard blood test sampling. While platforms like OStme (26) and TIMER (27) enable analyses of tumor-infiltrating cells and immune cell infiltration patterns. We prioritized establishing NHR as a practical clinical marker first. Future studies could explore the mechanistic relationships between NHR and the tumor immune microenvironment using these bioinformatics tools, which may reveal deeper insights into how systemic inflammation and lipid metabolism interact with local immune responses in the tumor microenvironment.

Several limitations should be acknowledged. The relatively small sample size, particularly in the validation cohort, may limit the generalizability of our findings. This also contributed to the less-than-ideal AUC values in the current manuscript; however, the disconnect between ROC analysis and clinical utility often reveals hidden biological complexity or data flaws rather than algorithm failure. It is worth noting that despite the suboptimal AUC values, the model shows significant clinical relevance in the age-stratified analysis.

In summary, this study emphasizes the age-dependent prognostic value of NHR in HCC postoperative recurrence, providing a new perspective on the relationship between inflammation, lipid metabolism, and aging in cancer progression. Creating a validated prognostic nomogram that includes NHR offers a practical tool for personalized risk assessment, especially for elderly patients. By addressing the heterogeneity of HCC through an integrated biomarker and considering age-specific effects, this study underscores the importance of age-stratified methods in HCC prognosis. It proposes a strategy for future research on inflammatory lipid markers in cancer and their clinical applications aimed at enhancing patient outcomes in this challenging disease.

## Data Availability

The raw data supporting the conclusions of this article will be made available by the authors, without undue reservation.
